# A structural equation model for assessment of links between changes in serum triglycerides, -urate, and -glucose and changes in serum Calcium, -magnesium and -phosphate in Type 2 diabetes and non-diabetes metabolism

**DOI:** 10.1186/1475-2840-10-116

**Published:** 2011-12-22

**Authors:** Lena Håglin, Lennart Bäckman, Birgitta Törnkvist

**Affiliations:** 1 Department of Public Health and Clinical Medicine, Family Medicine, Umeå University SE-901 87 Umeå, Sweden; 2 Department of Statistics, Umeå University, SE-901 87 Umeå, Sweden

## Abstract

**Background:**

This study investigates the associations between changes in serum Triglycerides (S-TG), -Urate (S-Urate), and -Glucose (S-Glu) and changes in serum Calcium (S-Ca), -Magnesium (S-Mg), and -Phosphate (S-P) in patients with type 2 diabetes compared with non-diabetic patients.

**Methods:**

The analysis is based on data collected from a secondary prevention population of women and men (W/M) at risk for cardiovascular disease (type 2 diabetes, 212/200; non-diabetes 968/703). The whole population (n = 2083) had a mean age of 51.0 (9.7) years and was stratified for sex and according to type 2 diabetes or non-diabetes. The patients were followed for, either half a year or one year and changes in risk factors were calculated from follow-up to baseline, the time when patients were admitted to the health center. The pattern of relationships was evaluated using a structural equation model.

**Results:**

Higher S-TG and S-Glu but lower S-Urate was revealed at baseline in type 2 diabetes women and men as compared to their counterparts, non-diabetes patients. Women with type 2 diabetes had higher S-Ca and lower S-Mg than non-diabetes women. Changes in S-Glu were associated with changes in S-Ca (+), baseline S-Ca (+), and S-Urate (-) in type 2 diabetes men. Changes in S-Urate were associated with changes in S-Mg (+) in type 2 diabetes women and non-diabetes men. In men with non-diabetes, changes in S-Glu were associated with changes in S-Mg (-). In women with non-diabetes, changes in S-Glu were associated with changes in S-P (-) and changes in S-Urate with changes in S-Ca (+).

**Conclusion:**

With respect to metabolic disturbances in non-diabetes and the awareness of risk for type 2 diabetes, changes in S-Glu and changes in S-Ca, S-Mg, and S-P should be considered as risk factors for cardiovascular disease. Increased early detection and corrections of high S-Ca, low S-Mg, and S-P in obese patients may improve their metabolism and reduce the risk of CVD in patients with type 2 diabetes.

**Trial registration number:**

ISRCTN: ISRCTN79355192

## Background

Distinguishing between metabolic disturbances in obese non-diabetic patients and type 2 diabetic patients provides information on how obesity related risk factors-including dyslipidemia, hyperglycemia, and hyperuricaemia-influence progression to type 2 diabetes. As magnesium is involved in glucose intolerance and S-Mg and incidence of type 2 diabetes are inversely associated, it is important to consider whether the serum levels of magnesium are connected to metabolic control [1]. The disposal rate of glucose in diabetes was related to fasting plasma magnesium concentrations after a standard glucose tolerance test [2]. Low serum magnesium may be a predictor of both metabolic syndrome and type 2 diabetes [3]. Low serum levels of phosphate are related to the risk of insulin resistance in a healthy population [4]. With chronic hypophosphatemia, the disturbance on post-receptor level indicates insulin resistance [5]. An experimental study suggested that phosphate depletion causes a high level of intracellular Ca in pancreatic beta cells, which inhibits ATP-production and results in low insulin secretion [6]. Glucose loads induce urinary excretion of both calcium and magnesium [7] due to decreased resorption in the proximal tubule [8]. This mechanism can be illustrated by the comparative effects from diuretic drugs, which may result in magnesuria and acidosis, reflected by an increased urinary excretion with or without changes in S-Mg [9,10].

This study tests whether there is a link between changes in serum Triglycerides (S-TG), -Urate (S-Urate), and -Glucose (S-Glu) and changes in serum Calcium (S-Ca), -Magnesium (S-Mg), or -Phosphate (S-P) over time and identifies if and how any associations are specific for either type 2 diabetes or the non-diabetes condition after stratification for sex. We tested the following three hypotheses (**Hn**) using the SEM.

**H1: **Changes in S-TG are related to baseline values of, and changes in, S-Ca, S-Mg, and S-P when controlling for age, baseline values of S-TG, and baseline values of, and changes in, S-Glu, BMI, SBP, and S-Chol.

**H2: **Changes in S-Urate are related to baseline values of, and changes in, S-Ca, S-Mg, and S-P when controlling for age, baseline values of S-Urate, and baseline values of, and changes in, S-Glu and BMI.

**H3: **Changes in S-Glu are related to baseline values of, and changes in, S-Ca, S-Mg, and S-P when controlling for age, baseline values of S-Glu, and baseline values of, and changes in, BMI.

The changes over time were measured at different time spans for different individuals at 0.5 or 1.0 years.

## Methods

### The Study Population

The study included 2083 patients (1180 women and 903 men) admitted to the Vindeln Health Center between 1984 and 1996. The patients were referred from primary care center and hospitals in the county and admitted to the center in groups of 30 for a four-week residential comprehensive activity: 64% were hypertensive's, 20% had type 2 diabetes, and 55% had a BMI > 30. In addition to the main diagnosis, around 50% had a second subsidiary diagnosis and the prevalence of multiple risk factors resembling metabolic syndrome were high. None of the patients included in the non-diabetes population had registered reduced glucose tolerance or type 2 diabetes. The characteristics of the patient population are described in Table 1. Of the 2083 patients at baseline, 744 were followed-up after 0.5 year (35.7%) and 1339 after 1.0 year (64.3%). The analysis is based on data collected on women and men (W/M) at risk for cardiovascular disease (type 2 diabetes, 212/200; non-diabetes 968/703). Written informed consent was obtained from the participants of this study. The Ethical Committee of North Sweden at the University of Umeå, Sweden, approved the protocol in November 22, 2006 (Dnr 05-177M). The study has been registered as a sub study to the Lifestyle intervention trial (no. ISRCTN79355192).

**Table 1:** Mean and standard deviation (sd) for baseline characteristics and variables included in the SEM.

	Type 2 diabetes			Non-diabetes			Women vs women^1^	Men vs men^2^
**Variables**	**Women n = 212**	**Men** **n = 200**	**p-value**	**Women** **n = 968**	**Men** **N = 703**	**p-value**	**p-value**	**p-value**

Age, yrs	51.3 (11.3)	53.5 (8.8)	0.0232	50.6 (9.9)	50.7 (9.1)	0.8117	0.4226	0.0001
BMI, kg/m^2^	32.3 (5.6)	30.3 (4.5)	< .0001	31.0 (5.7)	30.5 (4.9)	0.0447	0.0046	0.5246
SBP, mmHg	146 (19)	148 (19)	0.3778	146 (19)	149 (19)	0.0023	0.8778	0.3456
S-Chol, mmol/l	6.6 (1.3)	6.6 (1.5)	0.9024	6.7 (1.4)	6.7 (1.4)	0.6422	0.2132	0.2311
S-TG, mmol/l	2.8 (2.4)	3.2 (2.5)	0.0639	2.1 (1.2)	2.7 (2.0)	< .0001	< .0001	0.0079
S-Glu, mmol/l	10.7 (4.6)	10.8 (4.6)	0.8787	5.3 (1.1)	5.4 (1.3)	0.0029	< .0001	< .0001
S-Urate, mmol/l	298 (80)	322 (84)	0.0036	316 (72)	384 (72)	< .0001	0.0028	< .0001
S-Ca, mmol/l	2.37 (0.11)	2.34 (0.12)	0.0285	2.35 (0.10)	2.34 (0.09)	0.0063	0.0323	0.6119
S-Mg, mmol/l	0.78 (0.13)	0.81 (0.12)	0.0017	0.83 (0.12)	0.83 (0.13)	0.4164	< .0001	0.0610
S-P, mmol/l	1.05 (0.21)	0.97 (0.18)	< .0001	1.05 (0.21)	0.96 (0.20)	< .0001	0.9502	0.6015

Comparisons between type-2 diabetes and non-diabetes patients and for women with type 2 diabetes vs. women with non-diabetes and for men with type 2 diabetes vs. men with non-diabetes.

**1**/for comparisons between type 2 diabetes women and non-diabetes women

**2/**for comparisons between type 2 diabetes men and non-diabetes men

### The Life Style Intervention Program

The education and rehabilitation program at Vindeln Health Center (VHE-center) has been described earlier [11,12]. A problem-based learning perspective was established and the responsibility of planning and executing the activities was shared between patients and the staff. The staff consisted of a doctor, a nurse, physiotherapists, a dietician, and other important support staff.

During the first four weeks, 114 full-time hours were dedicated to food preferences, selections, physical exercise, and stress management. Half a year or one year after the program, the activities were repeated during a four-day revisit to the center. The objective of this follow-up was to establish functioning groups as a source to achieve changes and as a start for individual targets. The main focus was to combat risk of future cardiovascular disease (CVD). A refresher course was used to reinforce and revise the "home program".

### Assessments of Physical and Biochemical Variables

On the second day after arriving to VHE-center, systolic blood pressure (SBP; mmHg), body weight (kg), and height (cm) were recorded and body mass index (BMI) calculated (kg/m^2^). For every patient, a trained nurse measured blood pressure using a semi-automatic machine. Blood variables were analyzed on the serum sample according to the following standard routine methods at the Department of Clinical Chemistry, University Hospital, Umeå, Sweden.

Serum glucose (S-Glu) was determined using a hexokinase method (Boehringer Mannheim Diagnostica, Mannheim, Germany) on either Hitachi 717 or Hitachi 911. Serum cholesterol (S-Chol) was determined using an enzymatic method (Boehringer Mannheim Diagnostica, Mannheim, Germany) on either Hitachi 705 or Hitachi 717. Serum triglycerides (S-TG) were determined using enzymatic methods (Boehringer Mannheim Diagnostica, Mannheim, Germany) on Hitachi 717. Serum urate (S-Urate) was determined using an enzymatic Uricase method (Boehringer Mannheim Diagnostica, Mannheim, Germany) on Hitachi 705 or Hitachi 717.

Serum calcium (S-Ca) was determined using a complexometric method from Boehringer Mannheim on SMA II (Technicon) and on Hitachi 717 (after 1989). Up until 1989, serum magnesium was determined using the atomabsorption technique; after 1989, serum magnesium was determined using a colorimetrically-complex method with reagens from Boehring Mannheim on Hitachi 717. Serum phosphate (S-P) was determined using an ammonium-molybdate method (Boehringer Mannheim Diagnostica, Mannheim, Germany) on a Boehringer Mannheim (SMA II Technicon) and on Hitachi 717 (after 1989).

### Statistical Analysis

The study population was stratified according to sex and the diagnosis type 2 diabetes or non-diabetes. Repeated t-tests of differences in mean for nine variables at baseline were performed for women with type 2 diabetes compared to men with type 2 diabetes, for non-diabetes women compared to non-diabetes men, for women with type 2 diabetes compared to non-diabetes women and for men with type 2 diabetes compared to non-diabetes men.

**Figure 1:**
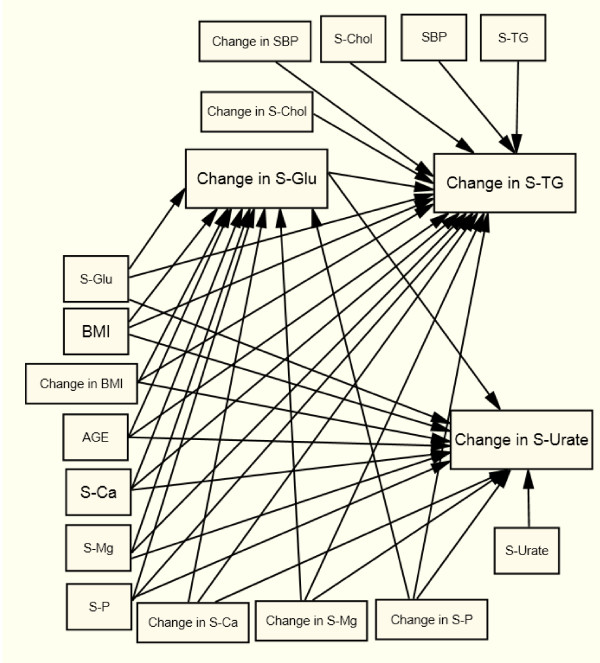
**The Structural Equation Model estimated for women with type 2 diabetes (X^2 ^= 21.8, df = 19, p = 0.294, RMSEA = 0.027, p-close = 0.786); men with type 2 diabetes (X^2 ^= 21.2, df = 19, p = 0.324, RMSEA = 0.024, p-close = 0.7896); women with non-diabetes (X^2 ^= 79.2, df = 19, p < 0,0001, RMSEA = 0,057, p-close = 0,166); and men with non-diabetes (X^2 ^= 59.9, df = 19, p < 0.0001, RMSEA = 0.055, p-close = 0.266)**.

The patients were followed for, either half a year or one year and changes in risk factors were calculated from follow-up to baseline, the time when patients were admitted to the health center. Structural Equation Modeling (SEM) was used to analyze the associations between changes in S-TG, S-Urate, S-Glu, and change in S-Ca, S-Mg, and S-P, with age, change in BMI, SBP, and serum cholesterol and baseline values as controlling variables (Figure 1). The model was estimated in one step for each of the four patient groups using the Generalized Least Squares (GLS) method [13].

Imputations were done with values from later time when available. Otherwise, average values were used for missing values. T-tests and chi^2^-tests were used to test for differences between patients with few relatively many missing values (0-1 and 5-8, respectively) at follow-up regarding sex, type 2 diabetes or non-diabetes, BMI at baseline, and age at admittance. We found no significant differences except for sex (p = 0.0443): 5% of the women and 3% of the men had 5-8 missing (85 patients out of 2083). No missing values were evident for the variables sex, age, and diagnosis (type 2 diabetes or non-diabetes); the other variables had missing values between 3% and 12%.

The changes over time were measured at different time spans for different individuals over 0.5 to 1.0 year and with imputations from 1.5, and 5 years. These differences were adjusted using a time variable (t = time to follow-up) in the equations. Model fit was tested with chi^2^- and RMSEA-tests. For every analysis, we used SAS version 9.2. (SAS Institute, Cary. NC). The figures were drawn with AMOS version 18.

## Results

### Baseline Data

A significantly higher S-TG and S-Glu levels and significantly lower S-Urate levels were seen for both women and men with type 2 diabetes compared to non-diabetes women and men. Women with type 2 diabetes had higher BMI and S-Ca and lower S-Mg than non-diabetes women (Table 1).

Comparing women and men at baseline within each diagnostic group reveals that women with type 2 diabetes had higher BMI, S-Ca, and S-P but lower S-Urate and S-Mg compared to men with type 2 diabetes. Non-diabetes women had lower SBP, S-TG, S-Glu, and S-Urate but higher S-Ca and S-P as compared to men with non-diabetes.

### Changes in Common for the Four Groups

**Figure 2:**
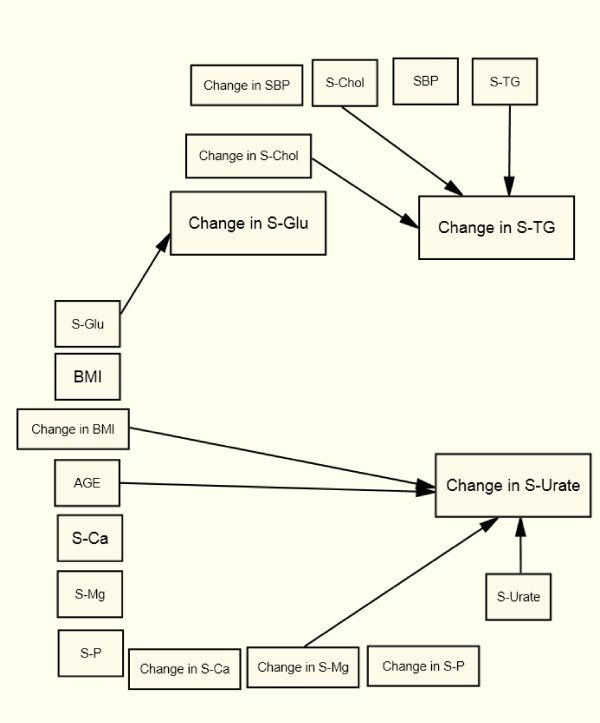
**Arrows indicate significant relations (p < 0.05) in the Structural Equation Model for women with type 2 diabetes**.

**Figure 3:**
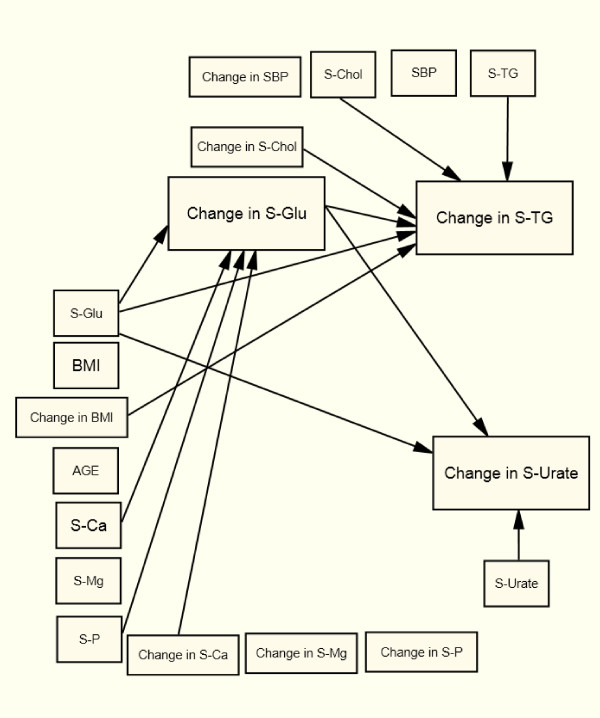
**Arrows indicate significant relations (p < 0.05) in the Structural Equation Model for men with type 2 diabetes**.

**Figure 4:**
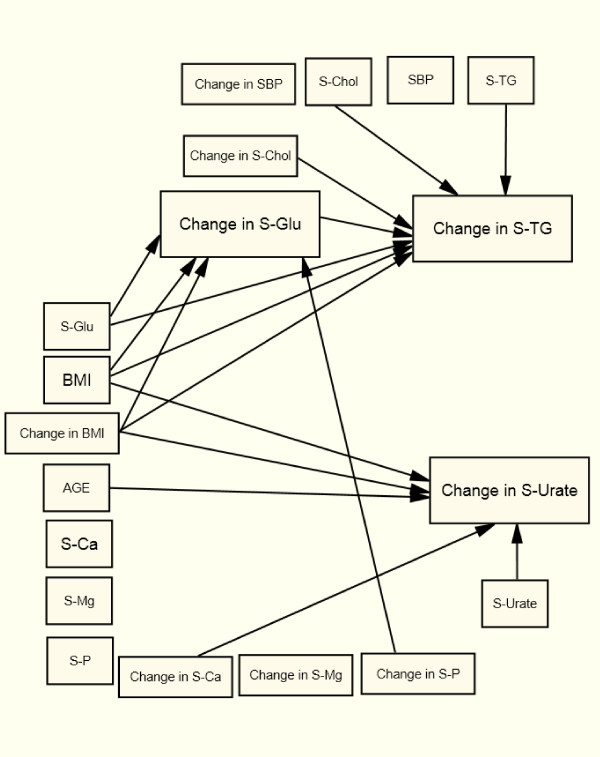
**Arrows indicate significant relations (p < 0.05) in the Structural Equation Model for women with non-diabetes**.

**Figure 5:**
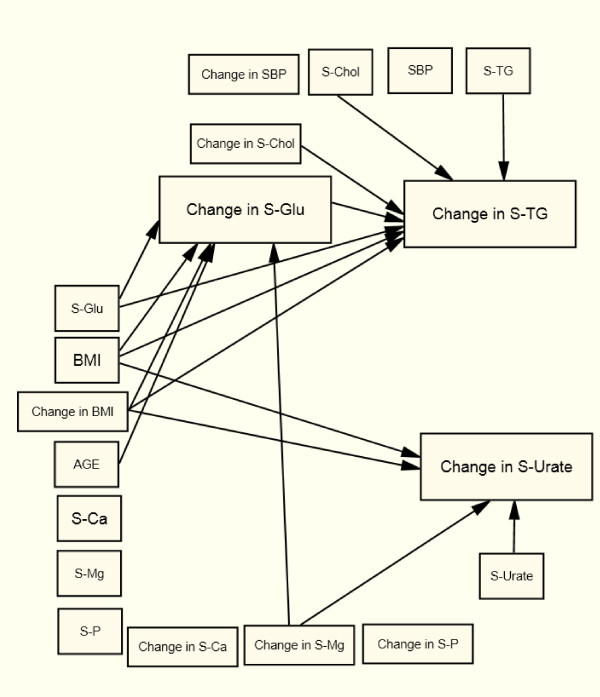
**Arrows indicate significant relations (p < 0.05) in the Structural Equation Model for men with non-diabetes**.

In all four sub-groups, changes in S-TG follow S-Chol (+) changes after controlling for baseline values, age, and changes in BMI and all the other metabolic variables included in the SEM (Table 2 and Figure 2, 3, 4, 5). In all four groups of patients, change in S-TG was related to baseline levels of both S-Chol (+) and S-TG (-), change in S-Urate was related to baseline S-Urate (-), and change in S-Glu was related to baseline S-Glu (-). After adjustments, no direct association was seen between changes in S-TG and baseline values or to changes in S-Ca, S-Mg, or S-P in any of the studied groups (Table 2 and Figure 2, 3, 4, 5).

**Table 2:** Estimated regression coefficients in SEM, with changes in S-TG as endogenous variable, for type 2 diabetes and non-diabetes women and men.

	Type 2 diabetes		Non-diabetes	
	**Women**	**Men**	**Women**	**Men**

**Variables**	**Par est**.	**Par est**.	**Par est**.	**Par est**.

Change in S-TG				
Age	0.015	-0.011	-0.001	-0.010
*Baseline*				
BMI	0.012	0.036	0.017**^c^**	0.032**^a ^**
SBP	-0.007	-0.006	-0.001	-0.002
S-Chol	0.230 **^b^**	0.537**^c^**	0.108**^c^**	0.446 **^c^**
S-TG	-0.515 **^c^**	-0.424 **^c^**	-0.468**^c^**	-0.665 **^c^**
S-Glu	-0.007	0.118 **^b^**	0.089 **^c^**	0.251 **^c^**
*Change*				
BMI	0.065	-0.228**^b^**	0.054**^c^**	0.071 **^b^**
SBP	-0.007	0.012	-0.001	-0.003
S-Chol	0.573 **^c^**	0.611 **^c^**	0.186 **^c^**	0.841 **^c^**
S-Glu	0.029	0.117 **^b^**	0.096 **^c^**	0.108**^a^**
*Baseline*				
S-Ca	-1.510	0.799	-0.021	0.819
S-Mg	0.292	-1.464	-0.253	0.086
S-P	0.523	-1.521	0.063	-0.453
*Change*				
S-Ca	0.223	0.299	0.271	-0.375
S-Mg	-1.431	-2.386	-0.370	-0.958
S-P	0.261	-1.231	0.189	-0.520

**R^2^**	**0.6658**	**0.3798**	**0.4272**	**0.5548**

At the bottom, R^2 ^are presented.

**a **= p < 0.05; **b **= p < 0.01; **c **= p < 0.001

### Diagnose Specific Associations

Change in S-Urate was directly associated with change in BMI (+), age (+) and change in S-Mg (+) in type 2 diabetes women (Figure 2).

Change in S-Urate was associated with baseline S-Glu and a change in S-Glu (-) in type 2 diabetes men (Figure 3). Change in S-Glu was directly related to baseline S-Ca (+) and S-P (-) and to a change in S-Ca (+) in type 2 diabetic men with an indirect effect on both changes in S-TG and changes in S-Urate (Figure 3).

In both women and men with non-diabetes, change in S-TG was related to baseline BMI (+) and S-Glu (+) and to changes in BMI (+) and changes in S-Glu (+) (Table 2 Figure 4 and 5). Change in S-Urate was related to BMI (+) at baseline and to changes in BMI (+) (Table 3 Figure 4 and 5). Change in S-Glu was related to BMI (+) at baseline and to change in BMI (+) (Table 4 Figure 4 and 5).

**Table 3:** Estimated regression coefficients in SEM, with changes in S-Urate as endogenous variable, for type 2 diabetes and non-diabetes women and men.

	Type 2 diabetes		Non - diabetes	
	**Women**	**Men**	**Women**	**Men**

**Variables**	**Par-est**.	**Par-est**	**Par est**.	**Par est**.

Change in S-Urate				
Age	1.273 **^c^**	0.097	0.785 **^c^**	-0.145
*Baseline*				
BMI	1.507	1.500	1.886 **^c^**	1.062**^a^**
S-Glu	-1.333	-4.764 **^c^**	0.476	3.087
S-Urate	-0.228 **^c^**	-0.304 **^c^**	-0.414 **^c^**	-0.314 **^c^**
*Change*				
BMI	4.122**^a^**	0.967	4.642 **^c^**	5.277**^c^**
S-Glu	-1.050	-5.073 **^c^**	1.027	-1.335
*Baseline*				
S-Ca	82.235	28.196	8.972	1.649
S-Mg	6.787	25.754	-6.680	22.986
S-P	-1.801	14.872	3.186	6.592
*Change*				
S-Ca	16.775	70.103	43.208**^a^**	22.217
S-Mg	133.5 **^b^**	52.535	30.075	50.062**^a^**
S-P	30.628	18.561	2.550	1.794

**R 2**	**0.2225**	**0.2859**	**0.2959**	**0.2068**

At the bottom, R^2 ^are presented.

**a **= p < 0.05; **b **= p < 0.01; **c **= p < 0.001

**Table 4:** Estimated regression coefficients in SEM, with changes in S-Glu as endogenous variable, for type 2 diabetes and non-diabetes women and men.

	Type 2 diabetes		Non - diabetes	
	**Women**	**Men**	**Women**	**Men**

**Variables**	**Par est**.	**Par est**.	**Par est**.	**Par est**.

Change in S-Glu				
Age	0.003	-0.002	0,004	0.016 **^c^**
*Baseline*				
BMI	-0.020	-0.085	0.029 **^c^**	0.052 **^c^**
S-Glu	-0.414 **^c^**	-0.692 **^c^**	-0.513 **^c^**	-0.499**^c^**
*Change*				
BMI	0.157	0.083	0.065**^c^**	0.078 **^c^**
*Baseline*				
S-Ca	5.479	7.251**^b^**	0.853	-0.378
S-Mg	1.528	-1.234	0.097	-0.391
S-P	-1.589	-3.507**^a^**	0.173	0.397
*Change*				
S-Ca	2.725	6.785**^a^**	0.524	-0.287
S-Mg	-1.297	-4.430	-0.482	-1.023 **^b^**
S-P	0.672	-2.775	-0.434**^a^**	-0.151

**R2**	**0.2225**	**0.5473**	**0.2320**	**0.2912**

At the bottom R^2 ^are presented.

**a **= p < 0.05; **b **= p < 0.01; **c **= p < 0.001

In non-diabetic women, change in S-Urate was directly related to age (+) and to a change in S-Ca (+), and a change in S-Glu was directly associated with a change in S-P (-), with an indirect effect on S-TG (Figure 4). In non-diabetic men, change in S-Glu was directly related to age (+) and directly to change in S-Mg (-), and a change in S-urate was directly related to change in S-Mg (+) (Figure 5).

### Gender Specific Results

In both type 2 diabetic women (Figure 2) and non-diabetic women (Figure 4) change in S-Urate was related to Age (+) and to change in BMI (+).

In both, type 2 diabetic men (Figure 3) and non-diabetic men (Figure 5) change in S-TG was related to baseline S-Glu (+) and to change in BMI (-) and S-Glu (+).

## Discussion

An association between the changes in S-TG, S-Urate, and S-Glu and BMI at both baseline and with change in BMI over time was shown in non-diabetic women and men, and these changes were not evident in women and men with type 2 diabetes except for an association between change in S-Urate and BMI change in women with type 2 diabetes. The strong association between change in S-Urate and BMI, shown in the obese non-diabetic condition in the present study, is a clinical finding described in connection with obesity and the metabolic syndrome [14]. The non-significant association, between changes in S-Urate and BMI at baseline for patients with type 2 diabetes, is an indication of the importance, of separating non-diabetes and obesity related metabolic disturbance from those within type 2 diabetes patients, in the assessment of CVD and mortality risk. Losing weight reduces CVD-risk, which, in the case of obesity, is partly explained by less body fat and reduced S-TG and S-Urate and improved S-Glu control. In the present study, a change in S-Glu was associated with a change in S-P (-) in non-diabetic women and with change in S-Mg (-) for non-diabetic men. This result could not have been due to weight loss as we adjusted for this in the SEM. These inverse associations had indirect effects on change in S-TG among non-diabetic patients. It has been postulated that the association between blood lipids and S-Mg are different in type 2 diabetes compared to non-diabetes [15]; however, no direct associations between changes in S-TG and changes in the three electrolytes could be seen.

A high S-Uric acid level in otherwise healthy individuals may be due to some oxidative stress and may predict type 2 diabetes [16]. In that study, high levels preceded the development of glucose intolerance and were not comparable to high S-Urate in type 2 diabetes.

The lower S-Urate at baseline in type 2 diabetes compared with non-diabetes and the inverse relation between changes in S-Urate and S-Glu in type 2 diabetes in our study could be explained by an increased resorption in proximal tubule and both insulin and S-Glu are involved in this regulation [17,18]. Recently, Oda and Kawai (2010) found that uric acid is positively associated with metabolic syndrome but negatively associated with diabetes in Japanese men [19]. These findings have been confirmed in a patient population where duration of type 2 diabetes, fasting blood glucose, and HbA1c were inversely related to the level of serum uric acid [20]. High glucose levels as indicated by HbA1c (a marker for the level of glucose over time) could be used to identify risk for diabetes [21]. Other metabolic markers (including using uric acid as a marker for changes in S-glucose) will give additional information about metabolic control. In pre-diabetic women, hyperuricemia was positively associated with level of S-Glucose [22].

In the present study, the inverse relation between changes in S-Glu and S-Urate over a short time in type 2 diabetic men, not reported earlier, suggests a metabolic disturbance of interest in understanding why high S-Urate in some studies does not increase CVD risk. Among women but not men with both type 2 diabetes and non-diabetes, change in S-Urate was positively associated with age.

Can changes in any of S-Ca, S-Mg, or S-P be linked to the inverse relation between changes in S-Urate and changes in S-Glu in type 2 diabetic men in the present study? The results indicate that when S-Glu changes, caused by S-Ca changes, an indirect and inverse effect on S-Urate was revealed. This association was positive, e.g., B-Glu increased with increasing S-Ca.

Changes in S-Glu were also associated with baseline S-Ca in type 2 diabetic men, which further gives strength to our hypothesis of links between energy metabolism and the three electrolytes. In addition, women with type 2 diabetes had higher S-Ca than non-diabetic women at baseline indicating some common disturbances in women and men with type 2 diabetes. On the contrary, S-Glu change was inversely related to baseline S-P in men with type 2 diabetes, indicating that a high S-P might cause a decrease in B-Glucose. With high S-P, secretion of insulin is normalized and peripheral glucose uptake improved [23]. These results indicate an involvement of both S-Ca and S-P in achieving glucose control.

S-Ca may be released from the surface of the bones and be responsible for the increase in glucose due to low S-P levels, which stimulate bone resorption. Whether an increase in calcium is a result from the disturbed glucose handling or from other mechanisms is not known, but it has been suggested that diabetic patients should be checked for hypercalcemia at appropriate intervals [24]. A signaling pathway for the link between energy metabolism and bone remodeling has been presented [25]. The correlations revealed between osteocalcin and insulin resistance might include changes in both S-P and S-Ca, and it would have been interesting to know if a high concentration of S-Ca together with high S-Glu is associated with lack of osteocalcin. The signaling pathway, with increase in leptin due to obesity and decrease in osteocalcin, may help regulate, primary or secondary, these ionic changes.

Earlier, we described that high S-Ca but low S-Mg without involvement from S-P can predict all-cause mortality in type 2 diabetes, although this is stronger for men than for women [26]. Other studies also describe high mortality with high S-Ca [27,28]. Women with type 2 diabetes had in addition to their lower S-Mg, significantly higher S-Ca compared to men with type 2 diabetes and non-diabetic women at baseline. It has been postulated that, parathyroid hormone mediates the direct effects from phosphate, calcium, vitamin D, and magnesium on metabolic syndrome in women but not men [29]. The ionic involvement is important to highlight as low S-Mg and low S-P and high S-Ca have implications for CVD risk in type 2 diabetes with or without disturbed glucose and urate metabolism.

It has been suggested that the metabolic disturbances in type 2 diabetes causes hypomagnesaemia [30,31]. Women with type 2 diabetes might lose more S-Mg than men do as indicated from low baseline values and from the strong response from the change in magnesium on change in S-Urate. A change in S-Mg associated with change in S-Urate, however, was also revealed in non-diabetic men.

With a decrease in S-Glu and concomitantly increase in S-Mg and decrease in S-Ca, the risk for CVD may be reduced even though S-Urate increases. An increase in S-Mg might have had an intracellular effect, which is important to consider in risk level determination, related to ischemia, catecholamine elevations, and insulin resistance [32,33]. This indicates that it is not enough to improve metabolic control with insulin or oral hypoglycaemic agents: it is also important to reverse hypomagnesemia [34]. A supplementation with magnesium may be needed to correct the low levels.

## In conclusion

The metabolic disturbances in non-diabetes condition is associated with changes in body weight, indicated from the findings of this study, of changes in only non-diabetic women and men but not in type 2 diabetic patients. In addition, changes in S-P (women) and S-Mg (men) were associated with changes in S-Glu (+) in the non-diabetic population. Thus, the low S-P in obesity might disturb the regulation of S-Glu in non-diabetes. This disturbance, in turn, might result in high S-Ca and low S-Mg in type 2 diabetes, maybe the two missing links in the study of CVD risk in this patient population. These associations are strengthened by the result of an association between changes in S-Urate and S-Mg (+) in women with type 2 diabetes, which is associated with change in S-Glu (-). In non-diabetics, low S-P should be considered as a risk factor for type 2 diabetes, while low S-Mg and high S-Ca should be considered as risk factors for CVD in type 2 diabetic patients.

## Abbreviations

BMI: Body Mass Index; CVD: Cardiovascular Disease; SBP: Systolic Blood Pressure; SEM: Structural Equation Modeling; S-Ca: Serum Calcium: S-Chol: Serum Cholesterol; S-Glu: Serum Glucose; S-Mg: Serum Magnesium; S-P: Serum Phosphate; S-TG: Serum Triglycerides; S-Urate: Serum Urate; VHE-center: Vindeln Health Center.

## Competing interests

The authors declare that they have no competing interests.

## Authors' contributions

LH planned and conceived of the study and participated in its design and had main responsibility in writing. LB carried out the statistical analysis and participated in the sequence alignment. BT conceived of the study and participated in its design and coordination and had responsibility for the design of the structural equation modeling and constructed all figure drafts in AMOS. BT also supported the draft of the manuscript. All authors read and approved the final manuscript.
